# Biological pathways leading to septo-optic dysplasia: a review

**DOI:** 10.1186/s13023-025-03541-6

**Published:** 2025-04-03

**Authors:** Ludovica Pasca, Davide Politano, Federica Morelli, Jessica Garau, Sabrina Signorini, Enza Maria Valente, Renato Borgatti, Romina Romaniello

**Affiliations:** 1 Department of Brain and Behavioral Sciences, University of Pavia, Pavia, Italy; 2 Child Neurology and Psychiatry Unit, IRCCS Mondino Foundation, Via Mondino 2, 27100 Pavia, Italy; 3 Developmental Neuro-Ophthalmology Unit, IRCCS Mondino Foundation, 27100 Pavia, Italy; 4 Service des Troubles du Spectre de l’Autisme et apparentés, Département de psychiatrie, Lausanne University Hospital (CHUV), Lausanne, Switzerland; 5 Department of Molecular Medicine, University of Pavia, Pavia, Italy; 6 Neurogenetics Research Center, IRCCS Mondino Foundation, Pavia, Italy

**Keywords:** Septo-optic dysplasia, SOD plus, Genetic pathways, Rare disease

## Abstract

**Background:**

The precise etiology of septo-optic dysplasia (SOD) remains elusive, to date a complex interaction between genetic predisposition and prenatal exposure to environmental factors is believed to come into play. Being SOD such a heterogeneous condition, disruption of many developmental steps in the early forebrain development might occur. The knowledge of genes possibly determining SOD phenotype should be improved, therefore in this review the authors attempt to highlight the genetic pathways and genes related to this clinical condition.

**Main body:**

Literature search was conducted and updated in November 2023, using PubMed and Google Scholar to identify primary research articles or case reports with available full text using the following search string “case reports,” “humans,” “septo-optic dysplasia,” “optic nerve hypoplasia,” with a recognized genetic diagnosis. Moreover, a review of genetic pathways with an involvement in SOD etiology was conducted. This review thus represents the authors’ perspective based on selected literature. The several pathways presented might be already associated to other disease phenotypes and interplay with genes and pathways known to have a role in SOD determination. Those pathways may converge and thus, the implicated genes may function as cascading regulators at multiple levels.

**Conclusion:**

The present data suggest that genes other than *HESX1, SOX2, SOX3*, and *OTX2* might be investigated in candidate individuals with a clinical diagnosis of SOD corresponding to the presence of at least two diagnostic criteria, particularly in the presence of additional syndromic anomalies.

## Introduction

### Definition

Septo-optic dysplasia (SOD), also known as de Morsier syndrome, is a congenital disorder belonging to the mid-line brain malformations group [1]. Prevalence has been estimated to be 1 in 10.000 live births [2]. Traditionally, SOD has been characterized by the association of a classic clinical-neuroradiological triad consisting of midline brain defects, hypoplasia of the optic nerves and/or chiasm, and hypotalamic-pituitary axis dysfunction [3]. The percentage of patients presenting all the above-mentioned features is about 30–47% [3]; currently, at least two out of three of such findings are required for a clinical diagnosis of SOD [4, 5]. If only one element of the triad is documented, it should be referred to as a distinct entity.

The typical midline malformation is represented by the absence or disruption of septum pellucidum, a thin transparent membrane located in the brain between the body and anterior horns of the lateral ventricles [5]. Other midline brain abnormalities described in SOD include: thinning or agenesis of corpus callosum and structural abnormalities of the hypothalamic-pituitary (HP) axis, namely hypoplasia of the pituitary infundibulum and/or gland, and ectopic location of the posterior pituitary [3].

When a malformation of cortical development (MCD) is associated to SOD, the term “SOD-plus” should be adopted, while the term “SOD-spectrum” should be used in cases presenting with wider range of congenital anomalies [3, 4], with an increasing gradient of severity.

### Etiology, pathogenesis and development

A combination of genetic predisposition and prenatal exposure to environmental factors leading to disruption is believed to come into play as the etiology of SOD [3]. Being SOD such a heterogeneous condition, disruption of many developmental steps from early patterning to neuronal specification and guidance of commissural axons might come into play. Indeed, involved structures in SOD do have different embryonic origin: pituitary gland, hypothalamus, optic nerves, and forebrain all develop from the anterior neural plate, with the neurulation process starting at the third week of gestation. Pituitary gland and optic nerves originate around the 4th–7th week of gestation, while the structure of corpus callosum differentiates as a commissural plate within the lamina terminalis during the 4th–5th week of gestation, with earliest callosal axons appearing at around 10 weeks of gestation and the achievement of the complete morphology at around 15 weeks of gestation [6]. Septum pellucidum formation is directly related to corpus callosum development and occur starting from 10 to 12 weeks of gestation [6, 7].

#### Vascular hypothesis

One of the most studied theories regarding the etiology of SOD portrays this complex neuro-ophthalmological syndrome as a vascular disruption sequence [8], resulting from a defect in blood circulation of the uterine-placental unit, the placental-fetal unit and/or the fetus itself [9]. In 1995 Lubinsky [9] defined SOD as a developmental anomaly supporting the hypothesis of a vascular disruption sequence affecting the proximal trunk of the anterior cerebral artery as the possible cause.

Young maternal age has been considered one of the most prominent factors associated to SOD since 1979, when Elster and McArney [10] first reported that mothers’ age of children with SOD was less than the average age of pregnancy. Later on, young maternal age and SOD have been extensively found to be associated in numerous studies [11, 12]. Similarly, a probable vascular etiology has been hypothesized for other congenital disorders [8], some of which co-occur with SOD, such as gastroschisis [13–15] and amniotic band syndrome [16–21]. A possible explanation is that young maternal age correlates with a higher rate of binge alcohol consumption, cigarette smoking and use of illicit drugs during pregnancy [22], well-known risk factors for fetal vascular disturbances.

Moreover, young maternal age might be associated with increased levels of estrogen and other endocrine disruptors that might have multiple effects, as in gastroschisis [23]. When used during pregnancy, some drugs with a vascular effect such as valproic acid, phencyclidine, phenylpropanolamine and cocaine [24, 25] are associated with SOD development in the fetus.

Finally, the increasing frequency of SOD, might underline a changing environmental exposure to exogenous predisposing factors [26].

A distinct epidemiology combining a strong decreased maternal age effect, an increased incidence in primagravidas independent of the age effect, and a low maternal body mass index [27] was interpreted as supporting a specific disorder instead of a spectrum of different clinical manifestations.

Recently, an updated vascular disruption-based model [7] incorporating new imaging, genetic and epidemiologic data has been proposed [26, 28] and relies on the hypothesis of ‘a SOD disruptive sequence with extension’. Namely, disruption of the primary proximal anterior cerebral artery trunk causes optic nerve hypoplasia and/or septum pellucidum defect; then, disruption can extend from optic nerve hypoplasia to the pituitary, or from the septal defects to the cortex.

#### Genetic hypothesis

At present, in the great majority of cases, a unique cause of SOD cannot be identified. The majority of SOD diagnoses seems to be sporadic. Only rare familial cases associated to autosomal recessive inheritance have been described [3]. Generally, less than 1% of all cases have been associated with mutations in the few known SOD genes: *HESX1 (MIM# 182230)*, identified in 1998 [26], and *SOX3*, *SOX2*, and *OTX2* being recognized subsequently, involved in different stages of eyes and midline structures embryonic development [29].

*HESX1* belongs to the family of homeobox genes, essential for early differentiation of the forebrain and adenohypophysis [29]. *SOX2 (MIM#206900), SOX3 (MIM#300123),* and *OTX2 (MIM#610125)* genes encode for transcription factors involved in regulation of other DNA regions that are crucial for early formation of different tissues. More specifically, *OTX2* and *SOX2* both play intricate roles in the embryonic development of the optic nerve [3]. *SOX3* gene encodes a member of the SOX family transcription factors involved in the regulation of embryonic development and in the determination of the cell fate; the encoded protein may act as transcriptional regulator after forming protein complexes [30]. With recent advances of next generation sequencing (NGS) techniques and their implementation in the clinical practice, many genes and genetic pathways have been studied extensively and have been associated with different SOD clinical phenotypes; in some cases, a genetic predisposition to vascular disturbances has been found as with *COL4A1 (MIM#180000)* and *COL4A2 (MIM#614519)*, whose alteration leads to vascular disruption sequences, particularly in the central nervous system (CNS), leading to CNS development perturbation ranging from slight white matter alterations to porencephaly, with rarer cases expressing a SOD-like phenotype [31].

### Clinical features

A wide clinical heterogeneity ranging from asymptomatic to very severe neurological and endocrinological involvement has been associated with SOD. The earliest clinical manifestations usually include neonatal signs of hypoglycemia and hyperbilirubinemia with the evidence of visual impairment of heterogeneous degree [3, 4, 32]. The onset of endocrine disorders is highly variable and central hypothyroidism (70%) is considered the most frequent, followed by growth hormone deficiency (55%), adrenal insufficiency (50%) and central diabetes insipidus (30%) [3]. Many different neuro-ophthalmological presentations are documented, with the most frequent clinical finding of abnormal eye movements, which usually can be appreciated by the first three months of life, especially in cases of SOD with bilateral optic nerve hypoplasia, and deficit of visual fixation and smooth pursuit [33]. The cognitive profile can range from normal intellectual abilities associated to neuropsychological fragilities to profound intellectual disability. Other neurodevelopmental disorders, beyond intellectual disability, have been described in SOD, such as autism spectrum disorder, and other less complex behavioral problems. Almost 30% of patients with SOD are known to have epilepsy, presenting either with infantile spasms, generalized tonic–clonic seizures, or myoclonic seizures [34]. Seizures secondary to metabolic disorder (hypoglycemia or hyponatremia) are also common in the first period of life [3]. Furthermore, drug resistant focal epilepsy is frequently observed in SOD-plus conditions [35]. Finally, sleep disorders with different severities may be part of the clinical picture and may be ascribed both to midline defects and to visual impairment [36].

### SOD plus syndrome

As already stated, SOD might be associated with other brain malformations and, in the presence of Malformations of cortical development (MCDs), the term SOD plus syndrome has been adopted [3, 35]. Among MCDs, schizencephaly, polymicrogyria, focal cortical dysplasia and nodular heterotopia are recurrent findings in SOD-plus cases. According to Barkovich classification [37], both unilateral and bilateral schizencephaly are reported in SOD-plus cases available in literature [3]. When SOD is associated to other brain abnormalities, a more complex and severe phenotype with poorer prognosis is to be expected [3]. See Table 1 for a summary of reported neuroradiological patterns of SOD-plus.

**Table 1:** Literature review of SOD-plus neuroradiological pattern

References	Number of patients	Associated brain malformation	Adopted SOD neuroradiological criteria
Miller et al. [38]	3	a. Right perisylvian polymicrogyria	a. Septum pellucidum agenesis Optic chiasm hypoplasia
		b. Right open-lip schizencephaly	b. Septum pellucidum agenesis Optic chiasm hypoplasia
		c. Left parietal polymicrogyria	c. Septum pellucidum agenesis Optic chiasm hypoplasia
Camino et al. [39]	1	Right frontal subependymal nodular heterotopia	Bilateral optic nerve hypoplasia Septum pellucidum agenesis
Kwak et al. [40]	1	Thickening of bilateral insular cortex	Septum pellucidum agenesis Optic nerve hypoplasia
Karatas et al. [34]	2	A. tetraventricular communicating hydrocephalus, atrophy of the left hemisphere and brain stem B. Porencephalic area in the right hemisphere	NA
Matushita et al. [41]	1	Polymicrogyria, involving insula, frontal and temporal lobes	NA
Trabacca et al. [42]	1	Right occipital cortical dysplasia	NA
Signorini et al. [4]	7	Polymicrogyria; Schizencephaly; aspecific abnormal cortical development	Olfactory bulb agenesis; cerebellar vermis hypoplasia
Labate et al. [43]	1	Bilateral perisylvian polymicrogyria	Septum pellucidum agenesis
Zoric et al. [44]	1	Left temporal lobe polymicrogyria	NA
Callie et al. [45]	13	Polymicrogyria (isolated/bilateral/ perysilvian/frontal)(47%) Left open-lip schizencephaly (29%) Schizencephaly with polymicrogyria at a distant site (18%) Grey matter heterotopia (35%) Transmantle cortical dysplasia (6%)	NA
Valenzuela et al. [46]	1	Frontal cortical dysplasia and agyria	NA
Gutierrez et al. [47]	1	Right fronto-temporal closed-lip schizencephaly Left fronto-parietal polymicrogyria	Septum pellucidum agenesis Corpus callosum hypoplasia Bilateral optic nerve, optic chiasm and pituitary stalk hypoplasia
Wang et al. [48]	1	Right open-lip schizencephaly Right midbrain hypoplasia Right oculomotor nerve hypoplasia	Absence of the septum pellucidum Bilateral optic nerve hypoplasia
Ouazzani et al. [49]	1	Closed-lip schizencephaly	NA

The association of SOD with polymicrogyria and nodular heterotopia supports the idea that SOD etiology might come from alterations of different stages at diverse timing in fetal neurodevelopment and cannot be explained by one isolated event, whether vascular or not [39].

The authors will focus on published studies reporting biological and genetic findings that might be responsible for determining SOD in order to highlight possible dysregulated genes and altered functional pathways leading to SOD. By searching for a better understanding of underlying biological and genetic pathways, it might be feasible to improve the diagnostic yield of the syndrome and shed light into new areas of research.

## Methods

### Literature search

Literature search was conducted and updated in November 2023, using PubMed and Google Scholar to identify primary research articles or case reports with available full text using the following search string “case reports,” “humans,” “septo-optic dysplasia,” “optic nerve hypoplasia,” with a recognized genetic diagnosis. Moreover, a review of genetic pathways with an involvement in SOD etiology was conducted. This review represents the authors’ perspective based on selected literature. Restrictions about the publication period were not set, and only documents published in peer-reviewed English journals were selected.

### Study selection

Included primary research articles or case report studies responded to the following inclusion criteria: presence of a clinical-radiological diagnosis according to the most recent SOD diagnostic criteria and a confirmed genetic diagnosis with alteration in genes other than the already well-recognized ones (*HESX1, SOX2, SOX3,* and *OTX2)*. Articles reporting on genetic and biological pathways implicated in SOD etiology were also included.

**Fig. 1:**
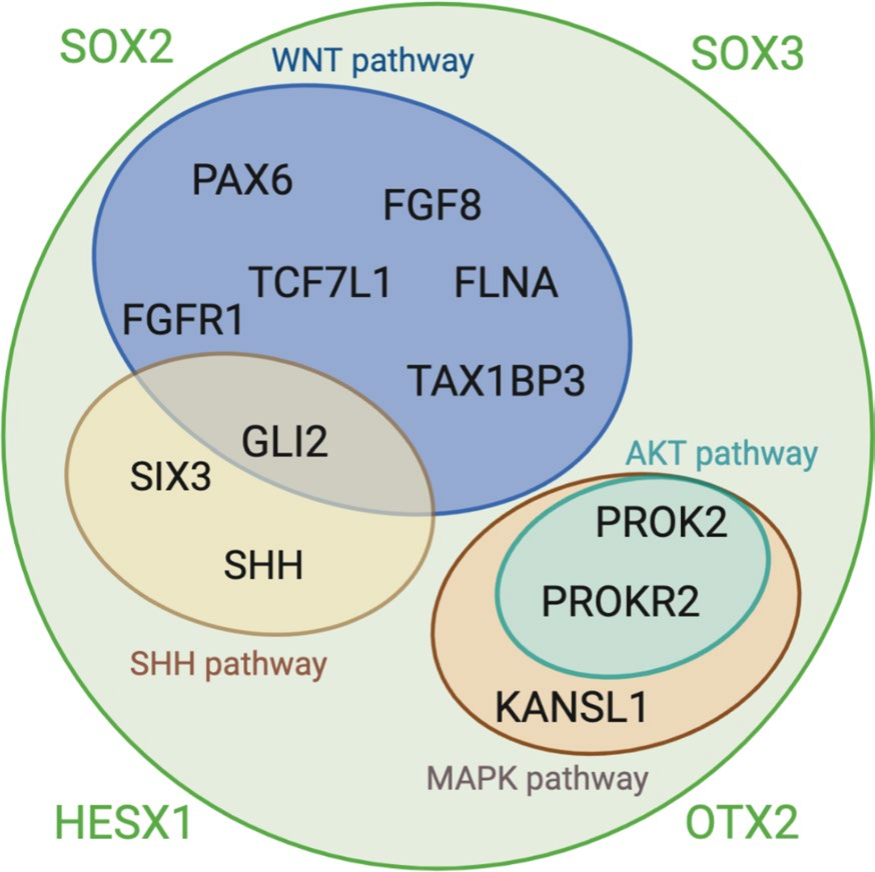
Genes and genetic pathways associated to SOD

Figure 1 resumes genetic pathways and related genes potentially implied in SOD pathogenesis as well as well-known SOD associated genes. As appreciable from the figure, the presented pathways often may converge and thus, the implicated genes may function as cascading regulators at multiple levels.

#### Genes associated with Septo-optic dysplasia

Twelve articles with genetic findings associated to SOD or SOD plus other than *HESX1, SOX2, SOX3*, and *OTX2* genes were considered. Whole exome sequencing (WES) was performed in nine out of twelve cases. CGH array was performed in two cases, karyotype was performed in two cases. Pathogenic variants and a genetic rearrangement were found in nine and three patients respectively [50–52]. SOD-plus patients [51, 53] carried complex genetic rearrangements identifiable at CGH array. Seven out of twelve patients also showed clinical features other than SOD diagnostic triad.

A detailed list of studies and genes is reported below. All available information about described variants are reported. See Table 2.

**Table 2:** Review of SOD patients’ neuroradiological, clinical and genetic findings with a genetic diagnosis other than *HESX1, SOX2, SOX3*, and *OTX2* genes were considered from the literature

First author	Year	Country	No. of patients	Performed genetic testing	Genetic diagnosis	ONH	Midline anomalies
Al-Salihi	2023	Qatar	1	Exome	TUBB mutation	Yes	Septum pellucidum agenesis, stretched and thin CC, hypoplastic splenium
Bravo	2012	USA	1	Karyotype	Interstitial deletion of the proximal portion of the long arm of chromosome 14	Yes	CC agenesis
Dhamija	2013	USA	1	CGH-array	Unbalanced 5;12 translocation	Yes	Septum pellucidum agenesis
Fernández-Marmiesse	2019	Spain	1	NGS (brain morphogenesis defects) negative, mRNA expression studies	Rare intronic variant c.6355+4_6355+5delAG in hemizygous state in the FLNA gene (reference sequence NM_001456.3)	Yes	CC hypoplasia
Gazdagh	2022	UK	1	Exome	NR2F1 initiation codon de novo missense variant	Yes	Absence of septum pellucidum, truncation of the rostrum of CC
Kawano-Matsuda	2020	Japan	1	NGS	ENG de novo variant	Yes (left)	No
Kinjo	2020	Japan	1	Exome	SMCHD1:p.Asp398Asn	Yes	No
Pasca	2023	Italy	1	Exome	SON:c.1069_1070delAG, (p.Arg357Thrfs*8)	Yes	Partial agenesis of CC and septum pellucidum
Reinstein	2015	Israel	1 family	Karyotype, exome	TAX1BP3 homozygous missense variant	Yes	CC and septum pellucidum agenesis
Reis	2022	USA	1	Exome	ARID1A:c.6625C > T(p.Gln2209*)	Yes	CC and septum pellucidum agenesis
Reyes-Capó	2018	USA	1	Exome	TUBA1A(NM_006009.4):c.715A > C(p.Thr239Pro)	Yes	Absence of septum pellucidum, truncation of the rostrum of CC
Singh	2004	USA	1	CGH-array	8q deletion/3p trisomy	Dentato-olivary dysplasia	deficient pituitary stalk, hypoplastic piyuitary gland, small optic nerves, absence of left olfactory bulb, absence of posterior half of CC
Slavotinek	2012	USA	1	exome	VAX1:p.Arg152Ser	Yes	Corpus callosum agenesis

First author	Hypothalamic/pituitary axis involvement	Malformation of cortical development	Other MRI abnormalities	Associated clinical features	ACMG classification
Al-Salihi	No	No	No	Facial dysmorphisms	NA
Bravo	Hypopituitarism	No	Ventriculomegaly, inferior vermis hypoplasia	Facial dysmorphisms, microcephaly, tracheomalacia, bronchopulmunary dysplasia, hypospadia	–
Dhamija	Hypopituitarism	No	No	Pre-axial polydactyly; patent ductus arteriosus, atrial and ventricular septal defects; facial dysmorphisms; hyperreflexia	–
Fernández-Marmiesse	Pituitary hypoplasia, growth rate drop (normal tests)	No	Delayed myelination	Interventricular septum hypertrophy	VUS (described as pathogenic PMID: 31234783)
Gazdagh	Growth hormone deficiency	No	Arnold-Chiari malformation	Iris coloboma, 2–3 toe syndactyly	Pathogenic (PMID: 26986877; 30945278; 34787370
Kawano-Matsuda	Hypothalamic pituitary dysfunction and hypoplasia	No	No	Strabismus, pulmunary AV fistulas	Pathogenic
Kinjo	Hypogonadotropic hypogonadism	No	No	No	Uncertain significance ( parents testing NA)
Pasca	Congenital hypothyroidism	No	No	Dysmorphisms, growth delay	Likely Pathogenic
Reinstein	Hypogonadotropic hypogonadism	No	No	Cardiomyopathy, facial dysmorphisms, macrocephaly	VUS, described as Pathogenic PMID:25645515
Reis	No	No	No	Hypoplastic big toe-nail, cleft palate, choanal atresia, sparse hair, and heart defects	Likely Pathogenic
Reyes-Capó	No	No	Band heterotopia and cerebellar hypoplasia	No	Likely Pathogenic
Singh	Hypopituitarism	Cortical dysplasia	Abnormal myelination, dento-olivry dysplasia	Cardiac malformation	–
Slavotinek	No	No	No	Microphthalmia and cleft lip/palate	Likely pathogenic

Reis et al. [54] described a single case carrying a de novo* ARID1A (MIM# 614607)* variant, *ARID1A*:c.6625C > T(p.Gln2209*), with SOD according to the presence of two out of the three diagnostic criteria (absence of septum pellucidum and corpus callosum, optic nerve hypoplasia). Systemic anomalies such as a hypoplastic big toe-nail, cleft palate, choanal atresia, sparse hair, and heart defects (ventricular septal defect and a patent foramen ovalis) were found in the described patient. *ARID1A* gene encodes a member of the SWItch/Sucrose Non Fermenting (SWI/SNF) complex. Variants in *ARID1A* are known to be responsible for Coffin-Siris syndrome, which is characterized by intellectual disability associated to agenesis or hypoplasia of the corpus callosum [54]. The identification of a role for *ARID1A* in SOD proposes the involvement of this gene and related pathway in this disorder, which was never reported before.

The study of Reinstein and colleagues [55] describes a family (two patients) carrying a homozygous missense variant in *TAX1BP3* gene, presenting agenesis of corpus callosum and absence of septum pellucidum, hypogonadotropic hypogonadism, bilateral optic disc hypoplasia in one of the two members, microcephaly, facial dysmorphisms, and severe dilated cardioyompthy. TAX1BP3 is highly expressed in developing heart and brain, encoding a small PDZ-containing protein implicated in the regulation of the Wnt/β-catenin pathway. Variants in the genes encoding Wnt/β-catenin pathway proteins (*TAX1BP3* e *TCF7L1*) have been hypothesized as causative of hypopituitary axis developmental defects with available studies on animal models [56].

A decreased expression of the NR2F1 protein has been described in association to SOD by Gazdgah et al. [57] in a patient with initiation codon de novo missense variant in *NR2F1* (MIM#615722) showing absence of septum pellucidum, truncation of the rostrum of corpus callosum and slender infundibulum, Chiari I malformation, developmental delay, seizures, optic atrophy and coloboma. NR2F1 protein is a nuclear hormone receptor and transcriptional regulator belonging to chicken ovalbumin upstream promoter transcription factors (COUP-TFs), which are orphan receptors of the steroid/thyroid hormone receptor superfamily [58]. Murine studies showed that *COUP-TFI* and *COUP*-*TFII* (*Nr2f1* and *Nr2f2*) genes are essential for early neural development and organogenesis. Moreover, Tang and colleagues [59] revealed that COUP-TFs are crucial for dorsalization of the eye and that *PAX6* and *OTX2*, described in SOD cases*,* are directly regulated by COUP-TFs.

A novel hemizygous out-of-frame deletion in *FLNA* (MIM#300321), c.6355+4_6355+5delAG, located in intron 38 of the gene, was found in a patient with neonatal hypoglycemia, optic nerve hypoplasia and dysmorphisms of corpus callosum described by Fernandez-Marmiesse and colleagues, thus presenting with two out of three SOD diagnostic criteria [60]. The patient also presented with interventricular septum hypertrophy and limb anomalies, well known findings in *FLNA*-associated syndrome. RNA studies showed that this variant results in the production of three aberrant *FLNA* transcripts, the most abundant of which results in the retention of intron 38. FLNA is implicated in signaling pathways that mediate organogenesis in multiple systems, involving the central nervous system during embryonic development [61]. The clinical picture of the reported patient potentially expands the phenotypic variability associated to *FLNA*.

A maternally inherited pathogenic *ENG* variant was found in a patient Hereditary hemorragic telangiectasia and optic nerve hypoplasia, pituitary gland hypoplasia and dysfunction, thus showing two out of three SOD diagnostic criteria [62]. *ENG* (MIM#187300) encodes for endoglin, which is a 180-kD glycoprotein expressed on endothelial cells, acting as an ancillary receptor for several transforming growth factors (TGF)-β superfamily ligands and modulating TGF-β1 and TGF-β3 responses [63]. This nonsynonymous variant was estimated to be pathogenic since previously reported in a patient with HHT [64] and functional prediction algorithms suggested that this variant might cause change of splice site. Kawano-Matsuda and colleagues [62] hypothesized that latent vascular insults during the fetal development might represent the common pathogenesis of congenital malformations both in the extremities and in midbrain that are found in SOD, and that the underlying microvascular abnormality of HHT during the development of cerebral midline may lead to SOD.

A heterozygous *TUBA1A* likely pathogenic variant, c.715A > C, was found by Reyes-Capò and colleagues [53] in a patient with corpus callosum agenesis, severe optic nerve hypoplasia, band heterotopia and cerebellar hypoplasia. Mutations in *TUBA1A* gene (MIM#611603), which encodes the microtubule-related protein α-tubulin, have been associated with a wide range of brain malformations including abnormalities of cortical development, hippocampi, basal ganglia, corpus callosum, cerebellum and brainstem. *TUBA1A* alterations have been described in two cases of optic nerve hypoplasia but variants in *TUBA8* (MIM#619840), also coding for an alpha tubulin as *TUBA1A*, have been linked to optic nerve hypoplasia [65]. Therefore, alpha tubulin components seem to be involved in both midline structures and optic nerve development.

Pasca and co-authors [66] recently reported an overlapping phenotype of Zhu-Tokita-Takenouchi-Kim syndrome (ZTTK) and SOD in a patient carrying a novel de novo *SON* gene (MIM#617140) heterozygous frameshift variant, c.1069_1070delAG, (p.Arg357Thrfs*8), and showing congenital hypothyroidism, psychomotor delay, dysmorphisms, growth delay, partial agenesis of septum pellucidum and corpus callosum, mild optic nerve, chiasm hypoplasia, and a small pituitary gland. The authors hypothesized that *SON* gene might have a regulatory function on the genes involved in SOD based on recent studies showing that *SON* gene haploinsufficiency in neuronal progenitors results in reduced mRNA expression and abnormal RNA splicing of multiple genes critical for neuronal migration, organization, brain development (e.g., *FLNA*, *TUBG1*, *PNKP*, *WDR62*, *PSMD3*, *HDAC6)*, and metabolism (e.g. *PCK2*, *PFKL*, *IDH2*, *ACY1*, *ADA*) causing neuronal migration defects and dendritic spine abnormalities [67]. Effects of *SON* haploinsufficiency on embryonic development are documented and result in several neurodevelopmental disorders associated with severe brain and eye malformations [68].

*SMCHD1* (MIM# 603457) encodes an epigenetic regulator that controls DNA methylation of multiple genomic loci [69]. Heterozygous *SMCHD1* mutations were identified in patients with Bosma arhinia microphthalmia syndrome (BAMS), an extremely rare syndrome whose clinical triad is represented by the absence of the nose, microphthalmia, and hypogonadotropic hypogonadism (HH). Kninjo and colleagues [69] described a patient with p.Asp398Asn variant in *SMCHD1* showing combined pituitary hormone deficiency (CPHD), optic nerve hypoplasia and thin retinal nerve fiber layer, therefore satisfying the criteria for SOD. Whole exome sequencing excluded additional variants in other HH/CPHD-causative genes. In vitro assays confirmed functional impairment of the described variant. These results suggest that the clinical consequences of *SMCHD1* mutations are broader than currently recognized, including septum pellucidum/corpus callosum hypoplasia, hearing loss, and cleft palate; HH, and eye anomalies have been documented in both conditions.

Slavotinek and colleagues described a patient bearing a homozygous VAX1 variant (p.Arg152Ser), predicted to be of LOF nature, in a proband of Egyptian origin with microphthalmia, small optic nerves, cleft lip/palate and corpus callosum agenesis, hence with a SOD phenotype [70]. VAX1 is essential for basal forebrain development, indeed it has been shown that Vax proteins function as activators of a dominant negative isoform of the Wnt signaling mediator TCF7L2, which is expressed throughout the developing forebrain [71]. The functional study conducted by the authors suggest that one mechanism whereby the mutation exerts its phenotypic effects is through the hyperactivation of Wnt signaling.

#### Genetic pathways associated to septo-optic dysplasia

##### Ras-RAF-EMK-ERK/mitogen-activated protein kinase signaling

The Ras-RAF-EMK-ERK/mitogen-activated protein kinase signaling pathway (ERK/MAPK pathway) finds its first actor in receptor-linked tyrosine kinases, which then triggers an intracellular phosphorylation cascade leading to phosphorylation and activation of ERK1/2-MAPK. The above results in different cellular events from proliferation, changes in cell differentiation, apoptosis and senescence [72]. *HRAS, KRAD* and *BRAF* are genes involved in ERK/MAPK pathway and participate in different steps of neurodevelopment processes including neural stem cell proliferation, neurogenesis, gliogenesis, and oligodendrocyte differentiation and myelination [73].

Germline mutations in components of ERK/MAPK pathway are known for being responsible of a set of syndromes defined as RASopathies [73]. Gualtieri and colleagues reported the association of SOD and RASopathies in the presence of *BRAF* gene mutations leading to a gain-of-function activation of MAPK pathway [74]. Activation of the MAPK pathway in the progenitors of the pituitary gland leads to abnormal terminal differentiation of hormone-producing cells, transient expansion of the pituitary stem cell pool followed by cell growth arrest and apoptosis leading to postnatal hypopituitarism. The authors also analyzed the expression pattern of BRAF during human embryonic development, and BRAF mRNA transcripts were localized throughout the neural tube, the retina, dorsal root ganglia, cranial nerves, and in the developing endocrine hypotalamo-pituitary axis, with prevalent expression in the ventral diencephalon and the Rathke’s pouch [74].

##### Wnt/β-catenin signaling

Wnt/β- catenin signaling pathways are recognized to have a major role in embryonic development, body axis patterning, and cell migration [75]. Specifically, the Wnt/β-catenin pathway plays critical roles in the proper patterning of the central nervous system from the earliest stages of neural development, driving neurodevelopmental processes such as CNS regionalization, neural progenitor differentiation, neuronal migration, dendrite development, synaptogenesis and adult neurogenesis [76]. The stability of β-catenin, which is a strong transcriptional activator, is critical for normal WNT/β-catenin signaling function [77]. In the absence of WNT ligands, β-catenin is phosphorylated and degraded, rendering the pathway inactive. β-Catenin can then translocate into the nucleus and interact with members of the T-cell factor/lymphoid enhancer factor family to activate the expression of target genes. In mammals, transcription factors like TCF7L1, TCF7L2*,* and LEF1, have a β- catenin–interacting domain at the N terminus. It is recognized that, in the absence of stable β-catenin, TCF/LEF factors can repress target genes of the pathway by the involvement of corepressors. It has been observed that alterations of the Wnt/β-catenin signaling pathway disrupt midbrain and hindbrain regionalization, and cause neural tube defects including conditions such as anencephaly, spina bifida, and craniorachischisis [76]. *TCF7L1,* for instance, is crucial to maintain normal expression of the hypothalamic signals involved in the induction and subsequent expansion of Rathke’s pouch progenitors, through its repressing activity of Wnt pathway [78]. As mentioned above, Vax proteins are activators of the canonical Wnt signaling mediator TCF7L2, having as an effect regulation of TCF7L2 target genes and Wnt signaling [71].

Moreover, *VAX1* apparently interacts with a downstream target of Wnt pathway that is PAX6 (MIM# 607108) [71]. Compound heterozygous mutations in PAX6 has been detected in two patients with complex brain and ocular malformations classifiable as SOD plus [79]. Heterozygous PAX6 mutation have been detected in patients presenting with various brain midline defects among which corpus callosum hypoplasia [80] but also ONH [81]. PAX6 is a transcription factor important for ocular development, by orchestrating the differentiation of different cell lines into the tissues constituting the eye, and for central nervous system embryonic development, through the government of cortical progenitor cell proliferation, neurogenesis, and neuronal layer formation. PAX6 also plays a crucial role in establishing dorso-ventral patterns, differentiating diverse CNS cell types, and defining boundaries along the anterior–posterior axis. Mutations of genes involved in Wnt/β-catenin signaling are the most represented in SOD diagnoses (*SOX2, SOX3, OTX2, TAX1BP3* and *TCF7L1).*

#### FGFR1 and FGF8 players

In humans, mutations in *FGF8* and *FGFR1* genes are known to cause congenital hypogonadotropic hypogonadism (CHH) without or with anosmia [82], Kallman syndrome, Hartsfield syndrome [83], holoprosencephaly and split hand/foot malformation [84]. Raivio and colleagues [85] have described a genetic overlap in patients with combined pituitary hormone deficiency CPHD/SOD carrying heterozygous mutations in *FGFR1* and *FGF8*, hypothesizing that mutations in genes generally associated with CHH/Kallman syndrome may also be associated with CPHD/SOD. Kallman syndrome is a developmental disease showing hypogonadism with anosmia but also absent or incomplete puberty, sexual immaturity, infertility, and midline defects [85]. In addition, *FGF8* mutations have been found to be associated with recessive holoprosencephaly, craniofacial defects, and hypothalamo-pituitary dysfunction [86].

During formation of the olfactory bulb and GnRH neurons, FGF8 acts mainly via FGFR1, i.e. one of the four FGF receptors [87] and its three isoforms (FGFR1-IIIa, FGFR1-IIIb and FGFR1-IIIc). Studies on mice carrying null mutations in FGFR1 revealed its fundamental role in early embryonic development, which reflects its involvement in neuralization and precursor proliferation [88]. Nuclear FGFR1 is required for neuronal differentiation and is expressed in Rathke’s pouch but also in the neuroepithelium where it regulates anterior–posterior patterning of telencephalon, being responsible for producing most of the frontal cortex [89]. FGF8 has two isoforms with distinct activity during brain development: FGF8a which exerts mainly a neural activity inducing the midbrain proliferation, and FGF8b, which is involved in mesoderm induction and differentiation [90]. Moreover, murine transcriptome data have identified members of the FGF8 signaling network during pituitary development [90]. Thus, FGF8 and FGFR1 might be early involved in processes leading to SOD.

##### PKB-AKT pathway

The phosphoinositide-3-kinase-protein kinase B/Akt (PI3K-PKB/Akt) pathway activation is controlled via a multistep process. Fully active PKB/Akt mediates numerous cellular functions including angiogenesis, metabolism, growth, proliferation, survival, protein synthesis, transcription, and apoptosis [91].

PI3K activates protein kinase B, also known as AKT, which represents a central node, being a positive regulator of several signaling pathways modulating cell proliferation, growth and survival, such as mTOR pathway. Particularly, in neurons located in the hippocampus, cerebral cortex and cerebellum, activation of the AKT/mTOR pathway seems to be essential for neuronal development and synapse formation [91]. The important function of PI3K in neurons has been demonstrated for its involvement in severe brain pathologies, such as developmentally-associated brain malformations, namely megalencephaly and focal cortical dysplasia [92]. Overall, studies on animal models and humans, indicate that PI3K/AKT is a central pathway for the integration of developmental signals that are necessary for brain development [93].

##### PROK2/PROKR2 players

PROK2 and its receptor PROKR2 are primarily expressed in the CNS, where they influence the olfactory bulb development and GnRH neural migration, but are produced in many other organs and tissues [94]. PROKR2 activation leads to mobilization of calcium, stimulation of phosphoinositide turnover and activation of p44/p42 mitogen-activated protein kinase [94].

Alterations of the PROK2/PROKR2 signaling pathway have been identified as causes of human Kallman syndrome. Specifically, PROK2/PROKR2 signaling has been recently demonstrated to be crucial for the tangential and radial migration of olfactory bulb interneurons [95]. Prok2 and prokr2 gene knockout mice both present abnormal GnRH neuron migration, agenesis, or hypoplasia of the olfactory bulbs, in association with hypogonadotropic hypogonadism [95]. Raivio and colleagues [85] searched for mutations in the PROK2/PROKR2 genes in patients with CPHD/SOD, identifying loss-of-function mutations in PROKR2 in unrelated CPHD/SOD probands but found the same variant (PROKR2 R268C variant) in heterozygous state in HH/Kallman syndrome patients, healthy first-degree relatives of Kallman syndrome probands, and in one of 250 healthy controls. With these findings, Raivio and colleagues [85] hypothesize that such involvement of PROK2/PROKR2 signaling pathway do not cause major midline defects per se, though it may act as a genes’ modifier.

##### SHH pathway

Sonic Hedgehog signaling (HSS) pathway is one fundamental network regulating key events of developmental processes. The pathway modulates the Shh protein, which constitutes one important signaling molecule implicated in the control of neurogenesis and neural patterning during CNS development [96, 97]. Shh signaling pathways is divided into canonical and non-canonical signaling, meaning a direct or indirect mediation of other pathways. Recent studies show that Shh regulates the development of the CNS through synergistic effect with temporal regulation appearing indispensable in determining the phenotype [96].

More specifically, Shh mediates ventral proliferation and differentiation of precursor cells [96] and neocortex development. In the CNS development of SHH gene knockout embryos of mice, early deficiency occurs in the midline structure and late deficiency includes the loss of distal limb structure, ciliary eye, the lack of ventral cell type in neural tube and the loss of spine and most ribs [98]. Conditional knockout of Shh in mice hypothalamus specifically resulted in a SOD phenotype [99]. The eye and pituitary develop in close proximity to the source of SHH in the anterior hypothalamus and depend on this signal for formation of the optic disc, from where the optic nerve exits the eye, and for coordinating pituitary morphogenesis. In support of a crucial role of *SHH*, *SOX2* and *SOX3*—two well documented SOD-associated genes—were shown to be dose-dependent regulators of *SHH* transcription that directly bind and activate a long-range *SHH* forebrain enhancer [99]. In humans, loss of function mutations in SHH are known to result in a variable clinical expression of holoprosencephaly phenotype [100], which results from imperfect separation of the cerebral hemispheres and craniofacial structures due to a reduction in SHH signaling from the prechordal plate. According to the timing and location of SHH signal disruption, a different phenotype might come out, possibly including SOD presentation.

GLI2 is an obligatory mediator of SHH signal transduction and is recognized among genes essential in pituitary formation*.* Loss-of-function mutations in the human *GLI2* gene (MIM# 610829) are associated with phenotypes belonging to holoprosencephaly (HPE) spectrum, whose primary features include defective anterior pituitary formation and pan-hypopituitarism, with or without overt forebrain cleavage abnormalities, and HPE-like mid-facial hypoplasia [101]. In the study of Soares Paulo and colleagues [102], a single heterozygous nonsense *SHH mutation* (p.Tyr175Ter) was found in a patient presenting with hypopituitarism and alobar HPE but its contribution to phenotype is uncertain as the in silico analysis did not predict it to be pathogenic. In the same study, a novel heterozygous missense variant in GLI2 (p.Leu761Phe) was found in a patient with SOD and CPHD; the same variant was found in the unaffected mother, with a possible explanation of incomplete penetrance. The resulting affected leucine residue is well conserved and lies in the GLI2 acetylation domain, which has been showed to be a key transcriptional checkpoint of Hedgehog signaling; in silico analysis predicted this variant to be damaging. Functional studies of the genetic variants described are needed to confirm genotype–phenotype correlation.

Finally, SHH signaling pathway resulted to be a key target of prenatal ethanol exposure and animal models with mutations in the Shh pathway genes showed a profound increase in the penetrance and severity of HPE when exposed to sub-teratogenic doses of ethanol [103].

## Conclusions

SOD typically has a low rate of genetic diagnosis. *The broad clinical heterogeneity that has driven researchers to interpret SOD as a spectrum of clinical manifestations rather than a specific entity, is counterbalanced by a specific epidemiology that has allowed the theorization of an updated vascular disruption-based model* [26]. Also, recent data on animal models and clinical reports show interesting insights into alterations in new and sometimes overlapping pathways. Not surprisingly, SOD and SOD-plus phenotype might derive from alterations in transcriptional pathways that intersect during brain development. Moreover, those pathways might be already associated to other disease phenotypes and interplay with genes and pathways known to have a role in SOD determination. Concurrent alterations in brain structures with different timing in development of SOD corroborates the hypothesis that the cause for this syndrome is related to an alteration in different stages of neurodevelopment and cannot be explained by one isolated event, whether vascular or not. When considering new plausible genes as responsible for SOD phenotype, other neurological and extra-neurological findings are usually found in addition to standard SOD diagnostic clues. The present data suggest that investigation for a genetic etiology should be warranted in individuals with a clinical diagnosis of SOD corresponding to the presence of at least two diagnostic criteria, particularly in the presence of additional syndromic anomalies. *Structural findings in non-genetic cases tend to be milder (e.g., thinning of the corpus callosum, ventriculomegaly, anomalies of the hippocampus,), and can be explained as secondary to thrombotic by-products of disruption transferred in the cerebrospinal fluid* [28]. Moreover, as suggested in previous literature [54], patients born from older, multigravida mothers should also represent good candidate for genetic testing.

## Data Availability

The datasets used and/or analyzed during the current study are available from the corresponding author on reasonable request.

## References

[1] De Morsier G. Studies on malformation of cranioencephalic sutures. III. Agenesis of the septum lucidum with malformation of the optic tract. Schweiz Arch Neurol Psychiatr. 1956;77(1–2):267–92. 13360148

[2] Webb EA, Dattani MT. Septo-optic dysplasia. Eur J Hum Genet. 2010;18(4):393–7. 19623216 10.1038/ejhg.2009.125 PMC2987262

[3] Ganau M, Huet S, Syrmos N, Meloni M, Jayamohan J. Neuro-ophthalmological manifestations of septo-optic dysplasia: current perspectives. Eye Brain. 2019;11:37–47. 10.2147/EB.S186307. 31695544 10.2147/EB.S186307 PMC6805786

[4] Signorini SG, Decio A, Fedeli C, Luparia A, Antonini M, Bertone C, et al. Septo-optic dysplasia in childhood: the neurological, cognitive and neuro-ophthalmological perspective. Dev Med Child Neurol. 2012;54(11):1018–24. 10.1111/j.1469-8749.2012.04404.x. 22924461 10.1111/j.1469-8749.2012.04404.x

[5] Sarwar M. The septum pellucidum: normal and abnormal. AJNR Am J Neuroradiol. 1989;10(5):989–1005. 2505543 PMC8335275

[6] Achiron R, Achiron A. Development of the human fetal corpus callosum: a high-resolution, cross-sectional sonographic study. Ultrasound Obstet Gynecol. 2001;18(4):343–7. 10.1046/j.0960-7692.2001.00512.x. 11778993 10.1046/j.0960-7692.2001.00512.x

[7] Siala S, Homen D, Smith B, Guimaraes C. Imaging of the septum pellucidum: normal, variants and pathology. Br J Radiol. 2023;96(1151):20221058. 10.1259/bjr.20221058. 37194993 10.1259/bjr.20221058 PMC10607410

[8] Lubinsky MS. Association of prenatal vascular disruptions with decreased maternal age. Am J Med Genet. 1997;69(3):237–9. 9096750 10.1002/(sici)1096-8628(19970331)69:3<237::aid-ajmg5>3.0.co;2-i

[9] van Gelder MMHJ, van Rooij IALM, Miller RK, Zielhuis GA, de Jong-van den Berg LTW, Roeleveld N. Teratogenic mechanisms of medical drugs. Hum Reprod Update. 2010;16(4):378–94. 20061329 10.1093/humupd/dmp052

[10] Elster AB, McAnarney ER. Maternal age re septo-opticdysplasia. J Pediatr. 1979;94(1):162–3. 758405 10.1016/s0022-3476(79)80392-3

[11] Murray PG, Paterson WF, Donaldson MDC. Maternal age in patients with septo-optic dysplasia. J Pediatr Endocrinol Metab. 2005;18(5):471–6. 15921176 10.1515/jpem.2005.18.5.471

[12] Atapattu N, Ainsworth J, Willshaw H, Parulekar M, MacPherson L, Miller C, et al. Septo-optic dysplasia: antenatal risk factors and clinical features in a regional study. Horm Res Paediatr. 2012;78(2):81–7. 22907285 10.1159/000341148

[13] Kamien B, Zankl A, Gabbett M. Septo-optic dysplasia and associations with amyoplasia and gastroschisis. Birth Defects Res Part A Clin Mol Teratol. 2010;88(6):497–501. 10.1002/bdra.20663 20589918

[14] Jordan MA, Montezuma SR. Septo-optic dysplasia associated with congenital persistent fetal vasculature, retinal detachment, and gastroschisis. Retin Cases Br Rep. 2015;9(2):123–6. 10.1097/ICB.0000000000000113 25397592

[15] Garvin J, Sampath V, Karody V. Gastroschisis complicated by septo-optic dysplasia: two distinct anomalies with a common origin. Am J Perinatol Rep. 2015;06(01):e15–7. 10.1055/s-0035-1563720 PMC4737633 26929863

[16] Pagon RA, Stephan MJ. Septo-optic dysplasia with digital anomalies. J Pediatr. 1984;105(6):966–8. 6502352 10.1016/s0022-3476(84)80092-x

[17] Faivre L, Amiel J, Ouachée-Chardin M, Geneviève D, Munnich A, Cormier-Daire V, et al. Septo-optic dysplasia and digital anomalies: another observation [1]. Am J Med Genet. 2002;108(3):247–8. 11891695 10.1002/ajmg.10267

[18] Orrico A, Galli L, Zappella M, Monti L, Vatti GP, Venturi C, et al. Septo-optic dysplasia with digital anomalies associated with maternal multidrug abuse during pregnancy. Eur J Neurol. 2002;9(6):679–82. 12453085 10.1046/j.1468-1331.2002.00473.x

[19] Stevens CA, Dobyns WB. Septo-optic dysplasia and amniotic bands: further evidence for a vascular pathogenesis. Am J Med Genet. 2004;125A(1):12–6. 14755460 10.1002/ajmg.a.20417

[20] Temtamy SA, Aglan MS, Ashour AM, El-Badry TH. Limb malformations with associated congenital constriction rings in two unrelated Egyptian males, one with a disorganization-like spectrum and the other with a probable distinct type of septo-optic dysplasia. Clin Dysmorphol. 2010;19(1):14–22. 19940763 10.1097/MCD.0b013e3283337d92

[21] Amiji IA, Mohamed UH, Rutashobya AG, Mngoya M, Schoenmann N, Naburi HE, et al. Septo-optic dysplasia with amniotic band syndrome sequence: a case report. J Med Case Rep. 2019;13(1):4–9. 31839004 10.1186/s13256-019-2306-2 PMC6913001

[22] Gladstone J, Levy M, Nulman I, Koren G. Characteristics of pregnant women who engage in binge alcohol consumption. CMAJ. 1997;156(6):789–94. 9084383 PMC1227041

[23] Lubinsky M. Gastroschisis and endocrine disruptors. Endocrine Disruptors. 2015;3(1): e1039688.

[24] Dominguez R, Aguirre Vila-Coro A, Slopis JM, Bohan TP. Brain and ocular abnormalities in infants with in utero exposure to cocaine and other street drugs. Am J Dis Child. 1991;145(6):688–95. 1709777 10.1001/archpedi.1991.02160060106030

[25] Fisher MC, Zeisel SH, Mar MH, Sadler TW. Septo-optic dysplasia as a manifestation of valproic acid embryopathy. Teratology. 2001;64(2):83–6. 11460259 10.1002/tera.1049

[26] Lubinsky M, Razavi E. Delineating septo-optic dysplasia. Birth Defects Res. 2022;114(20):1343–53. 10.1002/bdr2.2095. 36200678 10.1002/bdr2.2095

[27] Garcia-Filion P, Borchert M. Optic nerve hypoplasia syndrome: a review of the epidemiology and clinical associations. Curr Treat Options Neurol. 2013;15(1):78–89. 10.1007/s11940-012-0209-2. 23233151 10.1007/s11940-012-0209-2 PMC3576022

[28] Lubinsky M. By-products of vascular disruption carried in the CSF affect prenatal brain development. Birth Defects Res. 2022;114(15):847–54. 35775635 10.1002/bdr2.2064

[29] McCabe M, Alatzoglou K, Dattani M. Septo-optic dysplasia and other midline defects: the role of transcription factors: HESX1 and beyond. Best Pract Res Clin Endocrinol Metab. 2011;25(1):115–24. 10.1016/j.beem.2010.06.008. 21396578 10.1016/j.beem.2010.06.008

[30] Stevanovic M, Drakulic D, Lazic A, Ninkovic DS, Schwirtlich M, Mojsin M. SOX transcription factors as important regulators of neuronal and glial differentiation during nervous system development and adult neurogenesis. Front Mol Neurosci. 2021;14: 654031. 10.3389/fnmol.2021.654031. 33867936 10.3389/fnmol.2021.654031 PMC8044450

[31] Grego L, Pignatto S, Rassu N, Passone E, Cogo P, Lanzetta P. Optic nerve hypoplasia, corpus callosum agenesis, cataract, and lissencephaly in a neonate with a novel COL4A1 mutation. Case Rep Ophthalmol. 2019;10(3):424–30. 31966034 10.1159/000505017 PMC6959118

[32] Riedl S, Vosahlo J, Battelino T, et al. Refining clinical phenotypes in septo-optic dysplasia based on MRI findings. Eur J Pediatr. 2008;167(11):1269–76. 10.1007/s00431-007-0666-x. 18231810 10.1007/s00431-007-0666-x

[33] Borchert M. Reappraisal of the optic nerve hypoplasia syndrome. J Neuro Ophthalmol. 2012;32(1):58–67. 10.1097/wno.0b013e31824442b8. 10.1097/WNO.0b013e31824442b8 22330852

[34] Karatas H, Saygi S. Two cases of septo-optic dysplasia-plus syndrome with epilepsy and mirror hand movements. Epilepsy Behav. 2009;15(2):245–8. 10.1016/j.yebeh.2009.02.046. 19268717 10.1016/j.yebeh.2009.02.046

[35] AlKhateeb M, McLachlan R, Burneo J, Diosy D, Mirsattari S. Six adult patients with septo-optic dysplasia and drug-resistant epilepsy: clinical findings and course. Epilepsy Behav Case Rep. 2017;8:73–84. 10.1016/j.ebcr.2017.04.001. 29159066 10.1016/j.ebcr.2017.04.001 PMC5678750

[36] Yates JF, Troester MM, Ingram DG. Sleep in children with congenital malformations of the central nervous system. Curr Neurol Neurosci Rep. 2018;18(7):38. 10.1007/s11910-018-0850-6. 29789951 10.1007/s11910-018-0850-6

[37] Barkovich AJ, Guerrini R, Kuzniecky RI, Jackson GD, Dobyns WB. A developmental and genetic classification for malformations of cortical development: update 2012. Brain. 2012;135(Pt 5):1348–69. 10.1093/brain/aws019. 22427329 10.1093/brain/aws019 PMC3338922

[38] Miller SP, Shevell MI, Patenaude Y, Poulin C, O’Gorman AM. Septo-optic dysplasia plus: a spectrum of malformations of cortical development. Neurology. 2000;54(8):1701–3. 10.1212/wnl.54.8.1701. 10762523 10.1212/wnl.54.8.1701

[39] Camino R, Arjona A. Septo-optic dysplasia plus. Lancet Neurol. 2003;2(7):436. 10.1016/s1474-4422(03)00441-1. 12849124 10.1016/s1474-4422(03)00441-1

[40] Kwak JG, Jung S, Kwon SB, Hwang SH, Lee BC, Kwon KH. A patient with septo-optic dysplasia plus. J Neurol Sci. 2008;264(1–2):166–7. 10.1016/j.jns.2007.07.019. 17761198 10.1016/j.jns.2007.07.019

[41] Matushita JP Jr, Tiel C, Batista RR, Py M, Gasparetto EL. Septo-optic dysplasia plus: clinical presentation and magnetic resonance imaging findings. Arq Neuropsiquiatr. 2010;68(2):315–6. 10.1590/s0004-282x2010000200032. 20464308 10.1590/s0004-282x2010000200032

[42] Trabacca A, De Rinaldis M, Gennaro L, Losito L. Septo-optic dysplasia-plus and dyskinetic cerebral palsy in a child. Neurol Sci. 2012;33(1):159–63. 10.1007/s10072-011-0590-8. 21533562 10.1007/s10072-011-0590-8

[43] Labate A, Gambardella A, Quattrone A. Septo-optic dysplasia plus bilateral perisylvian polymicrogyria: a case report. Neurol Sci. 2013;34(8):1479–80. 10.1007/s10072-012-1227-2. 23124487 10.1007/s10072-012-1227-2

[44] Zoric L, Nikolic S, Stojcic M, Zoric D, Jakovljevic S. Septo-optic dysplasia plus: a case report. BMC Res Notes. 2014;7:191. 10.1186/1756-0500-7-191. 24678945 10.1186/1756-0500-7-191 PMC3976455

[45] Alt C, Shevell MI, Poulin C, Rosenblatt B, Saint-Martin C, Srour M. Clinical and radiologic spectrum of septo-optic dysplasia: review of 17 cases. J Child Neurol. 2017;32(9):797–803. 10.1177/0883073817707300. 28482731 10.1177/0883073817707300

[46] Infante-Valenzuela A, Camara-Lemarroy CR, Reyes-Mondragon AL, Muñiz-Landeros CE, Villarreal-Velazquez HJ. Septo-optic dysplasia plus diagnosed in adulthood. Neurol Sci. 2017;38(9):1705–7. 10.1007/s10072-017-2985-7. 28474147 10.1007/s10072-017-2985-7

[47] Gutierrez-Castillo A, Jimenez-Ruiz A, Chavez-Castillo M, Ruiz-Sandoval JL. Septo-optic dysplasia plus syndrome. Cureus. 2018;10(12): e3727. 10.7759/cureus.3727. 30800538 10.7759/cureus.3727 PMC6384050

[48] Wang CY, Ginat DT. Neuroimaging of septo-optic dysplasia-plus with midbrain hypoplasia and ophthalmoplegia. eNeurological Sci. 2020;19:100235. 10.1016/j.ensci.2020.100235. 10.1016/j.ensci.2020.100235 PMC7078351 32195380

[49] Ouazzani LCE, Jadib A, Laoudiyi D, Youssef S, Chbani K, Salam S, Ouzidane L. Dysplasie septo optique plus: à propos d’un cas [Septo optic dysplasia plus: about a case]. Pan Afr Med J. 2022;42:17. 10.11604/pamj.2022.42.17.33198. 35812255 10.11604/pamj.2022.42.17.33198 PMC9228923

[50] Dhamija R, Waltman L, Hoppman N, Kirmani S. Septo-optic dysplasia in a patient with an unbalanced 5;12 translocation. Pediatr Neurol. 2013;49(1):e2-3. 10.1016/j.pediatrneurol.2013.04.019. 23827435 10.1016/j.pediatrneurol.2013.04.019

[51] Singh V, Boesel CP, Baker P. Septo-optic dysplasia and dentato-olivary dysplasia in a case of 18q deletion/3p trisomy. Clin Neuropathol. 2004;23(1):28–33. 14986931

[52] Bravo EK, White ML, Olney AH, McAllister JL, Zhang YD. Novel proximal 14q deletion: clinical and diffusion tensor imaging tractography findings in a patient with lissencephaly, agenesis of the corpus callosum, and septo-optic dysplasia. AJNR Am J Neuroradiol. 2012;33(2):E16–8. 10.3174/ajnr.A2745. 22194387 10.3174/ajnr.A2745 PMC7964813

[53] Reyes-Capó DP, Chen F, Wilson B, Tarshish B, Ventura CV, Read SP, Negron CI, Berrocal AM. Aggressive posterior retinopathy of prematurity and a TUBA1A mutation inde Morsier syndrome. Ophthalmic Surg Lasers Imaging Retina. 2018;49(8):629–32. 10.3928/23258160-20180803-12. 30114309 10.3928/23258160-20180803-12

[54] Reis LM, Seese S, Maheshwari M, Basel D, Weik L, McCarrier J, University Of Washington Center For Mendelian Genomics, Semina EV. Novel genetic diagnoses in septo-optic dysplasia. Genes (Basel). 2022;13(7):1165. 10.3390/genes13071165. 35885948 10.3390/genes13071165 PMC9320703

[55] Reinstein E, Orvin K, Tayeb-Fligelman E, Stiebel-Kalish H, Tzur S, Pimienta AL, Bazak L, Bengal T, Cohen L, Gaton DD, Bormans C, Landau M, Kornowski R, Shohat M, Behar DM. Mutations in TAX1BP3 cause dilated cardiomyopathy with septo-optic dysplasia. Hum Mutat. 2015;36(4):439–42. 10.1002/humu.22759. 25645515 10.1002/humu.22759

[56] Osmundsen AM, Keisler JL, Taketo MM, Davis SW. Canonical WNT signaling regulates the pituitary organizer and pituitary gland formation. Endocrinology. 2017;158(10):3339–53. 10.1210/en.2017-00581. 28938441 10.1210/en.2017-00581

[57] Gazdagh G, Mawby R, Self JE, Baralle D, Deciphering Developmental Disorders Study. A severe case of Bosch–Boonstra–Schaaf optic atrophy syndrome with a novel description of coloboma and septo-optic dysplasia, owing to a start codon variant in the NR2F1 gene. Am J Med Genet A. 2022;188(3):900–6. 10.1002/ajmg.a.62569. 34787370 10.1002/ajmg.a.62569

[58] Takamoto N, Kurihara I, Lee K, Demayo FJ, Tsai MJ, Tsai SY. Haploinsufficiency of chicken ovalbumin upstream promoter transcription factor II in female reproduction. Mol Endocrinol. 2005;19(9):2299–308. 10.1210/me.2005-0019. 15890675 10.1210/me.2005-0019 PMC1198323

[59] Tang K, Xie X, Park JI, Jamrich M, Tsai S, Tsai MJ. COUP-TFs regulate eye development by controlling factors essential for optic vesicle morphogenesis. Development. 2010;137(5):725–34. 10.1242/dev.040568. 20147377 10.1242/dev.040568 PMC2827684

[60] Fernández-Marmiesse A, Pérez-Poyato MS, Fontalba A, Marco de Lucas E, Martínez MT, CaberoPérez MJ, Couce ML. Septo-optic dysplasia caused by a novel FLNA splice site mutation: a case report. BMC Med Genet. 2019;20(1):112. 10.1186/s12881-019-0844-5. 31234783 10.1186/s12881-019-0844-5 PMC6591933

[61] Zhang J, Neal J, Lian G, Hu J, Lu J, Sheen V. Filamin A regulates neuronal migration through brefeldin A-inhibited guanine exchange factor 2-dependent Arf1 activation. J Neurosci. 2013;33(40):15735–46. 10.1523/JNEUROSCI.1939-13.2013. 24089482 10.1523/JNEUROSCI.1939-13.2013 PMC3787497

[62] Kawano-Matsuda F, Shimada Y, Omotobara-Yabe T, Itonaga T, Maeda M, Maeda T, Yamaguchi T, Kosho T, Ihara K. A case of septo-optic dysplasia with hereditary hemorrhagic telangiectasia: a previously unrecognized combination of malformations. Clin Dysmorphol. 2020;29(1):49–52. 10.1097/MCD.0000000000000278. 30946035 10.1097/MCD.0000000000000278

[63] Toporsian M, Gros R, Kabir MG, Vera S, Govindaraju K, Eidelman DH, Husain M, Letarte M. A role for endoglin in coupling eNOS activity and regulating vascular tone revealed in hereditary hemorrhagic telangiectasia. Circ Res. 2005;96(6):684–92. 10.1161/01.RES.0000159936.38601.22. 15718503 10.1161/01.RES.0000159936.38601.22

[64] McDonald J, Bayrak-Toydemir P, Pyeritz RE. Hereditary hemorrhagic telangiectasia: an overview of diagnosis, management, and pathogenesis. Genet Med. 2011;13(7):607–16. 10.1097/GIM.0b013e3182136d32. 21546842 10.1097/GIM.0b013e3182136d32

[65] Hebebrand M, Hüffmeier U, Trollmann R, Hehr U, Uebe S, Ekici AB, Kraus C, Krumbiegel M, Reis A, Thiel CT, Popp B. The mutational and phenotypic spectrum of TUBA1A-associated tubulinopathy. Orphanet J Rare Dis. 2019;14(1):38. 10.1186/s13023-019-1020-x. 30744660 10.1186/s13023-019-1020-x PMC6371496

[66] Pasca L, Politano D, Cavallini A, Panzeri E, Vigone MC, Baldoli C, Abbate M, Kullmann G, Marelli S, Pozzobon G, Vincenzi G, Nacinovich R, Bassi MT, Romaniello R. A novel de novo heterozygous mutation in the SON gene associated with septo-optic dysplasia: a new phenotype. Neuropediatrics. 2023. 10.1055/a-2114-4387. 37343586 10.1055/a-2114-4387

[67] Ueda M, Matsuki T, Fukada M, Eda S, Toya A, Iio A, et al. Knockdown of Son, a mouse homologue of the ZTTK syndrome gene, causes neuronal migration defects and dendritic spine abnormalities. Mol Brain. 2020;13(1):80. 10.1186/s13041-020-00622-4. 32448361 10.1186/s13041-020-00622-4 PMC7245844

[68] Kim JH, Shinde DN, Reijnders MRF, Hauser NS, Belmonte RL, Wilson GR, et al. De novo mutations in SON disrupt RNA splicing of genes essential for brain development and metabolism, causing an intellectual-disability syndrome. Am J Hum Genet. 2016;99(3):711–9. 10.1016/j.ajhg.2016.06.029. 27545680 10.1016/j.ajhg.2016.06.029 PMC5011044

[69] Kinjo K, Nagasaki K, Muroya K, Suzuki E, Ishiwata K, Nakabayashi K, Hattori A, Nagao K, Nozawa RS, Obuse C, Miyado K, Ogata T, Fukami M, Miyado M. Rare variant of the epigenetic regulator SMCHD1 in a patient with pituitary hormone deficiency. Sci Rep. 2020;10(1):10985. 10.1038/s41598-020-67715-x. 32620854 10.1038/s41598-020-67715-x PMC7335161

[70] Slavotinek AM, Chao R, Vacik T, Yahyavi M, Abouzeid H, Bardakjian T, Schneider A, Shaw G, Sherr EH, Lemke G, Youssef M, Schorderet DF. VAX1 mutation associated with microphthalmia, corpus callosum agenesis, and orofacial clefting: the first description of a VAX1 phenotype in humans. Hum Mutat. 2012;33(2):364–8. 10.1002/humu.21658. 22095910 10.1002/humu.21658 PMC3401628

[71] Hallonet M, Hollemann T, Pieler T, Gruss P. Vax1, a novel homeobox-containing gene, directs development of the basal forebrain and visual system. Genes Dev. 1999;13(23):3106–14. 10.1101/gad.13.23.3106. 10601036 10.1101/gad.13.23.3106 PMC317183

[72] Giménez N, Martínez-Trillos A, Montraveta A, Lopez-Guerra M, Rosich L, Nadeu F, Valero JG, Aymerich M, Magnano L, Rozman M, Matutes E, Delgado J, Baumann T, Gine E, González M, Alcoceba M, Terol MJ, Navarro B, Colado E, Payer AR, Puente XS, López-Otín C, Lopez-Guillermo A, Campo E, Colomer D, Villamor N. Mutations in the RAS-BRAF-MAPK-ERK pathway define a specific subgroup of patients with adverse clinical features and provide new therapeutic options in chronic lymphocytic leukemia. Haematologica. 2019;104(3):576–86. 10.3324/haematol.2018.196931. 30262568 10.3324/haematol.2018.196931 PMC6395334

[73] Kim YE, Baek ST. Neurodevelopmental aspects of RASopathies. Mol Cells. 2019;42(6):441–7. 10.14348/molcells.2019.0037. 31250618 10.14348/molcells.2019.0037 PMC6602148

[74] Gualtieri A, Kyprianou N, Gregory LC, Vignola ML, Nicholson JG, Tan R, Inoue SI, Scagliotti V, Casado P, Blackburn J, Abollo-Jimenez F, Marinelli E, Besser REJ, Högler W, Karen Temple I, Davies JH, Gagunashvili A, Robinson ICAF, Camper SA, Davis SW, Cutillas PR, Gevers EF, Aoki Y, Dattani MT, Gaston-Massuet C. Activating mutations in BRAF disrupt the hypothalamo-pituitary axis leading to hypopituitarism in mice and humans. Nat Commun. 2021;12(1):2028. 10.1038/s41467-021-21712-4. 33795686 10.1038/s41467-021-21712-4 PMC8016902

[75] Komiya Y, Habas R. Wnt signal transduction pathways. Organogenesis. 2008;4(2):68–75. 10.4161/org.4.2.5851. 19279717 10.4161/org.4.2.5851 PMC2634250

[76] Mulligan KA, Cheyette BN. Neurodevelopmental perspectives on Wnt signaling in psychiatry. Mol Neuropsychiatry. 2017;2(4):219–46. 10.1159/000453266. 28277568 10.1159/000453266 PMC5318929

[77] Liu J, Xiao Q, Xiao J, Niu C, Li Y, Zhang X, Zhou Z, Shu G, Yin G. Wnt/β-catenin signalling: function, biological mechanisms, and therapeutic opportunities. Signal Transduct Target Ther. 2022;7(1):3. 10.1038/s41392-021-00762-6. 34980884 10.1038/s41392-021-00762-6 PMC8724284

[78] Gaston-Massuet C, McCabe MJ, Scagliotti V, Young RM, Carreno G, Gregory LC, Jayakody SA, Pozzi S, Gualtieri A, Basu B, Koniordou M, Wu CI, Bancalari RE, Rahikkala E, Veijola R, Lopponen T, Graziola F, Turton J, Signore M, Mousavy Gharavy SN, Charolidi N, Sokol SY, Andoniadou CL, Wilson SW, Merrill BJ, Dattani MT, Martinez-Barbera JP. Transcription factor 7-like 1 is involved in hypothalamo-pituitary axis development in mice and humans. Proc Natl Acad Sci USA. 2016;113(5):E548–57. 10.1073/pnas.1503346113. 26764381 10.1073/pnas.1503346113 PMC4747739

[79] Glaser T, Jepeal L, Edwards JG, Young SR, Favor J, Maas RL. PAX6 gene dosage effect in a family with congenital cataracts, aniridia, anophthalmia and central nervous system defects. Nat Genet. 1994;7(4):463–71. 10.1038/ng0894-463. 7951315 10.1038/ng0894-463

[80] Sisodiya SM, Free SL, Williamson KA, Mitchell TN, Willis C, Stevens JM, Kendall BE, Shorvon SD, Hanson IM, Moore AT, van Heyningen V. PAX6 haploinsufficiency causes cerebral malformation and olfactory dysfunction in humans. Nat Genet. 2001;28(3):214–6. 10.1038/90042. 11431688 10.1038/90042

[81] Azuma N, Yamaguchi Y, Handa H, Tadokoro K, Asaka A, Kawase E, Yamada M. Mutations of the PAX6 gene detected in patients with a variety of optic-nerve malformations. Am J Hum Genet. 2003;72(6):1565–70. 10.1086/375555. 12721955 10.1086/375555 PMC1180317

[82] Young J, Xu C, Papadakis GE, Acierno JS, Maione L, Hietamäki J, Raivio T, Pitteloud N. Clinical management of congenital hypogonadotropic hypogonadism. Endocr Rev. 2019;40:669–710. 10.1210/er.2018-00116. 30698671 10.1210/er.2018-00116

[83] Palumbo P, Petracca A, Maggi R, Biagini T, Nardella G, Sacco MC, Di Schiavi E, Carella M, MicaleCastori LM. A novel dominant-negative FGFR1 variant causes Hartsfield syndrome by deregulating RAS/ERK1/2 pathway. Eur J Hum Genet. 2019;27:1113–20. 10.1038/s41431-019-0350-4. 30787447 10.1038/s41431-019-0350-4 PMC6777633

[84] Villanueva C, Jacobson-Dickman E, Xu C, Manouvrier S, Dwyer AA, Sykiotis GP, Beenken A, Liu Y, Tommiska J, Hu Y, et al. Congenital hypogonadotropic hypogonadism with split hand/foot malformation: a clinical entity with a high frequency of FGFR1 mutations. Genet Med. 2015;17:651–9. 10.1038/gim.2014.166. 25394172 10.1038/gim.2014.166 PMC4430466

[85] Raivio T, Avbelj M, McCabe MJ, Romero CJ, Dwyer AA, Tommiska J, Sykiotis GP, Gregory LC, Diaczok D, Tziaferi V, Elting MW, Padidela R, Plummer L, Martin C, Feng B, Zhang C, Zhou QY, Chen H, Mohammadi M, Quinton R, Sidis Y, Radovick S, Dattani MT, Pitteloud N. Genetic overlap in Kallmann syndrome, combined pituitary hormone deficiency, and septo-optic dysplasia. J Clin Endocrinol Metab. 2012;97(4):E694–9. 10.1210/jc.2011-2938. 22319038 10.1210/jc.2011-2938 PMC3319178

[86] McCabe MJ, Gaston-Massuet C, Tziaferi V, Gregory LC, Alatzoglou KS, Signore M, Puelles E, Gerrelli D, Farooqi IS, Raza J, Walker J, Kavanaugh SI, Tsai PS, Pitteloud N, Martinez-Barbera JP, Dattani MT. Novel FGF8 mutations associated with recessive holoprosencephaly, craniofacial defects, and hypothalamo-pituitary dysfunction. J Clin Endocrinol Metab. 2011;96(10):E1709–18. 10.1210/jc.2011-0454. 21832120 10.1210/jc.2011-0454 PMC3417283

[87] Linscott ML, Chung WCJ. TET1 regulates fibroblast growth factor 8 transcription in gonadotropin releasing hormone neurons. PLoS ONE. 2019;14(7): e0220530. 10.1371/journal.pone.0220530. 31361780 10.1371/journal.pone.0220530 PMC6667164

[88] Klimaschewski L, Claus P. Fibroblast growth factor signalling in the diseased nervous system. Mol Neurobiol. 2021;58(8):3884–902. 10.1007/s12035-021-02367-0. 33860438 10.1007/s12035-021-02367-0 PMC8280051

[89] Borello U, Cobos I, Long JE, McWhirter JR, Murre C, Rubenstein JL. FGF15 promotes neurogenesis and opposes FGF8 function during neocortical development. Neural Dev. 2008 14;3:17. 10.1186/1749-8104-3-17. Erratum in: Neural Develop. 2008 Nov 5;3: 31.. McWhirter, John R [added]. 10.1186/1749-8104-3-17 PMC2492847 18625063

[90] Kumar V, Goutam RS, Park S, Lee U, Kim J. Functional roles of FGF signaling in early development of vertebrate embryos. Cells. 2021;10(8):2148. 10.3390/cells10082148. 34440915 10.3390/cells10082148 PMC8391977

[91] Hemmings BA, Restuccia DF. PI3K-PKB/Akt pathway. Cold Spring Harb Perspect Biol. 2012;4(9): a011189. 10.1101/cshperspect.a011189. 22952397 10.1101/cshperspect.a011189 PMC3428770

[92] Jansen LA, Mirzaa GM, Ishak GE, O’Roak BJ, Hiatt JB, Roden WH, Gunter SA, Christian SL, Collins S, Adams C, Rivière JB, St-Onge J, Ojemann JG, Shendure J, Hevner RF, Dobyns WB. PI3K/AKT pathway mutations cause a spectrum of brain malformations from megalencephaly to focal cortical dysplasia. Brain. 2015;138(Pt 6):1613–28. 10.1093/brain/awv045. 25722288 10.1093/brain/awv045 PMC4614119

[93] Sánchez-Alegría K, Flores-León M, Avila-Muñoz E, Rodríguez-Corona N, Arias C. PI3K signaling in neurons: a central node for the control of multiple functions. Int J Mol Sci. 2018;19(12):3725. 10.3390/ijms19123725. 30477115 10.3390/ijms19123725 PMC6321294

[94] Martin C, Balasubramanian R, Dwyer AA, Au MG, Sidis Y, Kaiser UB, Seminara SB, Pitteloud N, Zhou QY, Crowley WF Jr. The role of the prokineticin 2 pathway in human reproduction: evidence from the study of human and murine gene mutations. Endocr Rev. 2011;32(2):225–46. 10.1210/er.2010-0007. 21037178 10.1210/er.2010-0007 PMC3365793

[95] Valdes-Socin H, Rubio Almanza M, Tomé Fernández-Ladreda M, Debray FG, Bours V, Beckers A. Reproduction, smell, and neurodevelopmental disorders: genetic defects in different hypogonadotropic hypogonadal syndromes. Front Endocrinol (Lausanne). 2014;5:109. 10.3389/fendo.2014.00109. 25071724 10.3389/fendo.2014.00109 PMC4088923

[96] Yang C, Qi Y, Sun Z. The role of sonic hedgehog pathway in the development of the central nervous system and aging-related neurodegenerative diseases. Front Mol Biosci. 2021;8: 711710. 10.3389/fmolb.2021.711710. 34307464 10.3389/fmolb.2021.711710 PMC8295685

[97] Odent S, Atti-Bitach T, Blayau M, Mathieu M, Aug J, de Delezo AL, Gall JY, Le Marec B, Munnich A, David V, Vekemans M. Expression of the Sonic hedgehog (SHH ) gene during early human development and phenotypic expression of new mutations causing holoprosencephaly. Hum Mol Genet. 1999;8(9):1683–9. 10.1093/hmg/8.9.1683. 10441331 10.1093/hmg/8.9.1683

[98] Echevarría-Andino ML, Allen BL. The hedgehog co-receptor BOC differentially regulates SHH signaling during craniofacial development. Development. 2020;147(23):dev189076. 10.1242/dev.189076. 33060130 10.1242/dev.189076 PMC7758635

[99] Zhao L, Zevallos SE, Rizzoti K, Jeong Y, Lovell-Badge R, Epstein DJ. Disruption of SoxB1-dependent Sonic hedgehog expression in the hypothalamus causes septo-optic dysplasia. Dev Cell. 2012;22(3):585–96. 10.1016/j.devcel.2011.12.023. 22421044 10.1016/j.devcel.2011.12.023 PMC3306593

[100] Society for Maternal-Fetal Medicine (SMFM); Monteagudo A. Holoprosencephaly. Am J Obstet Gynecol. 2020;223(6):B13–B16. 10.1016/j.ajog.2020.08.178. 10.1016/j.ajog.2020.08.178 33168217

[101] Roessler E, Du YZ, Mullor JL, Casas E, Allen WP, Gillessen-Kaesbach G, Roeder ER, Ming JE, Ruiz i Altaba A, Muenke M. Loss-of-function mutations in the human GLI2 gene are associated with pituitary anomalies and holoprosencephaly-like features. Proc Natl Acad Sci USA. 2003;100(23):13424–9. 10.1073/pnas.2235734100. 14581620 10.1073/pnas.2235734100 PMC263830

[102] Paulo SS, Fernandes-Rosa FL, Turatti W, Coeli-Lacchini FB, Martinelli CE Jr, Nakiri GS, Moreira AC, Santos AC, de Castro M, Antonini SR. Sonic Hedgehog mutations are not a common cause of congenital hypopituitarism in the absence of complex midline cerebral defects. Clin Endocrinol (Oxf). 2015;82(4):562–9. 10.1111/cen.12565. 25056824 10.1111/cen.12565

[103] Hong M, Krauss RS. Cdon mutation and fetal ethanol exposure synergize to produce midline signaling defects and holoprosencephaly spectrum disorders in mice. PLoS Genet. 2012;8(10): e1002999. 10.1371/journal.pgen.1002999. 23071453 10.1371/journal.pgen.1002999 PMC3469434

